# Wnt/β-Catenin Signaling and Resistance to Immune Checkpoint Inhibitors: From Non-Small-Cell Lung Cancer to Other Cancers

**DOI:** 10.3390/biomedicines11010190

**Published:** 2023-01-12

**Authors:** Satoshi Muto, Akio Enta, Yoshiyuki Maruya, Sho Inomata, Hikaru Yamaguchi, Hayato Mine, Hironori Takagi, Yuki Ozaki, Masayuki Watanabe, Takuya Inoue, Takumi Yamaura, Mitsuro Fukuhara, Naoyuki Okabe, Yuki Matsumura, Takeo Hasegawa, Jun Osugi, Mika Hoshino, Mitsunori Higuchi, Yutaka Shio, Kazuyuki Hamada, Hiroyuki Suzuki

**Affiliations:** Department of Chest Surgery, Fukushima Medical University, Fukushima 960-1295, Japan

**Keywords:** immune checkpoint inhibitors, tumor escape, immunomodulation, tumor microenvironment, beta catenin, Wnt signaling pathway, cancer, immunotherapy, non-small-cell lung cancer

## Abstract

Lung cancer is the leading cause of cancer-related deaths worldwide. The standard of care for advanced non-small-cell lung cancer (NSCLC) without driver-gene mutations is a combination of an anti-PD-1/PD-L1 antibody and chemotherapy, or an anti-PD-1/PD-L1 antibody and an anti-CTLA-4 antibody with or without chemotherapy. Although there were fewer cases of disease progression in the early stages of combination treatment than with anti-PD-1/PD-L1 antibodies alone, only approximately half of the patients had a long-term response. Therefore, it is necessary to elucidate the mechanisms of resistance to immune checkpoint inhibitors. Recent reports of such mechanisms include reduced cancer-cell immunogenicity, loss of major histocompatibility complex, dysfunctional tumor-intrinsic interferon-γ signaling, and oncogenic signaling leading to immunoediting. Among these, the Wnt/β-catenin pathway is a notable potential mechanism of immune escape and resistance to immune checkpoint inhibitors. In this review, we will summarize findings on these resistance mechanisms in NSCLC and other cancers, focusing on Wnt/β-catenin signaling. First, we will review the molecular biology of Wnt/β-catenin signaling, then discuss how it can induce immunoediting and resistance to immune checkpoint inhibitors. We will also describe other various mechanisms of immune-checkpoint-inhibitor resistance. Finally, we will propose therapeutic approaches to overcome these mechanisms.

## 1. Introduction

Lung cancer is the leading cause of cancer-related deaths worldwide, with approximately 1.8 million deaths in 2020 [1]. Up to half of lung-cancer patients, including those undergoing surgical resection, will unfortunately develop disease recurrence [2].

The current standard of care for advanced non-small-cell lung cancer (NSCLC) is molecular-targeted agents, chemotherapy, and immune checkpoint inhibitors. Molecular-targeted agents have improved the outcome of advanced NSCLC cases [3]. However, their indications are limited to patients with specific driver-gene mutations. Therefore, the development of immune checkpoint inhibitors has been promoted. Initially, anti-programmed death-1 (PD-1) antibodies [4,5,6] or anti-programmed death ligand-1 (PD-L1) monotherapy [7,8] was used, but combinations with chemotherapy [9,10,11,12,13] or anti-cytotoxic T-lymphocyte-associated protein-4 (CTLA-4) antibodies [14,15,16] have improved outcome, and have now been approved for the treatment of advanced NSCLC.

The therapeutic effects of immune checkpoint inhibitors are characterized by an impressive durable response. However, even in NSCLC patients with high PD-L1 expression who would benefit from anti-PD-1 or anti-PD-L1 antibodies, only approximately half of them achieve long-term responses to immune checkpoint inhibitors [9,10,11,12,13,14,15]. There is an urgent need to achieve therapeutic effects in patients who display resistance to immune checkpoint inhibitors. Therefore, it is necessary to elucidate the specific molecular mechanisms associated with resistance to immune checkpoint inhibitors.

Recently, oncogenic-signaling molecules such as Wnt/β-catenin, which have long been known to be essential for cancer-cell survival and proliferation, have attracted attention as potential resistance mechanisms to immune checkpoint inhibitors [17,18,19]. Several such resistance mechanisms have already been reviewed elsewhere [20,21,22]. However, most of these studies have been conducted on cancers other than NSCLC. In this review, we will summarize findings on immune-checkpoint-inhibitor-resistance mechanisms in NSCLC and other cancers, focusing on the Wnt/β-catenin signaling pathway. First, we will review the molecular biology of Wnt/β-catenin signaling, and then discuss how this pathway can induce immunoediting and resistance to immune checkpoint inhibitors. We will also examine Wnt/β-catenin signaling in the context of various other mechanisms of immune checkpoint inhibitor resistance. Finally, we will propose therapeutic approaches to overcome this resistance mechanism.

## 2. Wnt Pathways in Cancer

### 2.1. Canonical Wnt Pathway

The Wnt signaling pathway is a critical signal-transduction cascade between cell-surface molecules and the cytoplasm. This pathway mediates cell division, stem-cell pluripotency during embryonic development, and immune-cell development [23,24,25]. In addition to these important roles, Wnt signaling is critical for T-cell differentiation and functions [26]. The Wnt protein was discovered in 1982 [23], and, to date, humans are known to have at least 19 different Wnt proteins [27]. All these Wnt proteins have 23 or 24 cysteine residues, and the carboxy-terminal region may determine the specificity [28]. Regulation of β-catenin by Wnt is referred to as canonical Wnt signaling. In this pathway, glycogen synthase kinase-3β (GSK-3β) phosphorylates β-catenin, under the actions of the axin and adenomatous-polyposis-coli (Apc) complexes in the absence of Wnt-ligand binding. Marked by this phosphorylation, β-catenin is rapidly degraded by proteolysis. However, when Wnt ligand binds to a co-receptor consisting of Frizzled and the low-density lipoprotein receptor-related protein-5/6 (LRP5/6) receptor (in the signal “on-state”), the resulting activated Frizzled receptor acts via Dishevelled protein and axin to suppress GSK-3β [29,30]. As a result, β-catenin escapes degradation, and accumulates in the cytoplasm and nucleus. Once in the nucleus, β-catenin can associate with the DNA-binding T-cell factor (Tcf)/lymphoid enhancer-binding factor (Lef) transcription factors, and promote expression of a diverse set of Wnt-responsive genes by displacing Groucho transcriptional receptor proteins [30]. These Wnt-responsive genes include a set of genes involved in cell proliferation. *C-MYC* is one such Wnt-responsive gene. C-MYC regulates aerobic glycolysis, alanine-serine-cysteine transporter 2 (ASCT2) levels, and glutathione production [31,32,33,34]. The canonical Wnt pathway not only increases gene expression related to cancer-cell proliferation, but also acts on cancer-cell metabolism.

### 2.2. Non-Canonical Wnt Pathways

Although we mainly focus on canonical Wnt signaling (the Wnt/β-catenin signaling pathway) in this review, non-canonical Wnt pathways are also of note. In these pathways, Wnt binds to another subset of Frizzled receptors. Most of them are reported to be Frizzled6, which promotes cancer-cell invasion, metastasis, survival, and cell growth through G-protein activation [35]. Frizzled receptors and receptor tyrosine kinase-like orphan receptors (RORs) or receptor tyrosine kinase (RYK) coreceptors can activate Wnt/planar cell polarity (PCP), Wnt/receptor tyrosine kinase (RTK), and Wnt/Ca^2+^ signaling cascades [36]. Wnt/PCP signaling regulates actin cytoskeletal dynamics and cell movement. Its activation can support the invasion and metastasis of cancer cells. Wnt/RTK signaling through receptors such as ROR1, ROR2, and RYK can activate the phosphatidylinositol-3 kinase-protein kinase B (PI3K-AKT) signaling pathway [37]. Activated receptors in these non-canonical Wnt signaling cascades can increase inositol triphosphate (IP3) and diacylglycerol (DAG) levels. IP3 induces cytosolic Ca^2+^ elevation from the endoplasmic reticulum and activates protein kinase C (PKC). PKC activates nuclear factor kappa-B (NF-κB) and nuclear factor of activated T cells (NFAT), which are both transcription factors known to be activated in cancer cells and immune cells [38]. Therefore, these non-canonical Wnt pathways also play important roles in cancer growth and progression.

### 2.3. The Wnt/β-Catenin Pathway and Cancer

Numerous studies have reported associations between Wnt signaling and cancer progression. The first report, in 1987, identified the WNT factor gene as an oncogene in a mouse mammary cell line [39]. Mutations in β-catenin genes can reportedly lead to oncogenesis via activation of the Wnt/β-catenin signaling pathway [40,41]. As mentioned above, APC is an important factor in the regulation of the Wnt/β-catenin signaling pathway. It is well known that an *APC* gene mutation is the cause of familial adenomatous polyposis and hereditary-colon-cancer syndrome [42]. Splicing in *APC* and in-frame deletions in Catenin beta 1 (*CTNNB1*) leading to oncogenic Wnt pathway activation were found in 96% of colorectal-cancer tumors [43]. Gene mutations in *Axin1* or *Axin2* and deletions of *GSK3B* can lead to a reduction in the β-catenin degrading complex, and activate the Wnt/β-catenin signaling pathway [44,45,46]. Immunohistochemistry experiments have demonstrated that the overexpression of β-catenin was associated with poor overall survival in patients with stage IA–IIA squamous-cell lung cancer [47] and decreased progression-free survival in patients with ovarian cancer [48]. Additionally, β-catenin overexpression was associated with increased chemoresistance. Glutathione, resulting from the activation of the Wnt/β-catenin pathway, is associated with ovarian-cancer-cell chemoresistance [49], and is important for cancer-stem-cell maintenance [50]. In a lung adenocarcinoma cell line, Wnt/β-catenin signaling exhibited chemoresistance mechanisms via cancer-stem-cell properties [51]. While these oncogenic characteristics and metabolic modulations of the Wnt/β-catenin signaling pathway in cancer progression have been well established, their relationships with cancer immune-escape and cancer immunoediting mechanisms have been studied extensively only in recent years.

## 3. The Wnt/β-Catenin Pathway Is a Tumor-Intrinsic Mechanism of Resistance to Immune Checkpoint Inhibitors

### 3.1. Tumor-Intrinsic Mechanisms of Immune-Checkpoint-Inhibitor Resistance

For an anti-cancer immune response to be generated, each step of the cancer-immunity cycle must function in sequence [52]. However, the intrinsic characteristics of cancer cells can inhibit these steps in the cancer-immunity cycle.

First, if the tumor is not sufficiently immunogenic, an immune response will not occur. Immune cells recognize tumor neoantigens, and trigger an immune response. Therefore, tumor neoantigens are considered important for immunogenicity. Promising results have been reported for melanoma with vaccine-therapy development using personal neoantigens [53]. Although not a perfect biomarker, because of the other steps of the cancer-immunity cycle, there is an association between the tumor mutational burden and a therapeutic response to immune checkpoint inhibitors [14,54]. Microsatellite instability or mismatch-repair deficiency correlates with increased neoantigens. The efficacy of an anti-PD-1 antibody in patients with high microsatellite instability or mismatch-repair deficiency also suggests the importance of neoantigens in the immune response to cancer [55,56].

Second, major-histocompatibility-complex (MHC) deficiency is one of the resistant mechanisms against immune checkpoint inhibitors. Because presentation of cancer antigens is primarily caried out by MHC class I, many cancers attempt to evade immunity by suppressing MHC class I protein expression. MHC class I expression is reportedly associated with cancer prognosis [57]. Deletions of β_2_-microglobulin (B2M), which constitutes MHC class I, and transporters associated with antigen processing 1 (TAP1) and TAP2, which transport cancer antigens, have also been previously reported [58,59]. Deletion of MHC class I expression during cancer treatment has also been described, suggesting that it is caused by tumor evolution because of immunological pressure [60,61]. It has been reported that the combination of tumor-mutation burden and MHC class I loss can more accurately predict the response to immune-checkpoint-inhibitor treatment [62]. Mutations in *B2M* have also been demonstrated to be an acquired resistance mechanism against immune checkpoint inhibitors [63].

T cells recognizing cancer antigens on MHC class I release interferon-γ (IFN-γ), which in response activates the Janus kinase (JAK)-signal transducer and activator of transcription (STAT) pathway, and induces PD-L1 expression on the cancer-cell surface. PD-L1 is indeed part of one of the cancer-cell immune-escape mechanisms, but the dysfunction of this tumor-intrinsic IFN-γ signaling pathway is also an immune-checkpoint-inhibitor-resistance mechanism [21]. Mutations in the IFN-γ signaling pathway were frequently observed in non-responders to immune checkpoint inhibitors [63,64,65,66]. Comprehensive analyses using CRISPR also showed that genes related to the IFN-γ signaling pathway are involved in resistance mechanisms against immunotherapy [67,68,69,70].

Oncogenic signals can also affect immune-escape mechanisms in a variety of ways, as well as cancer-cell proliferation (Table 1). *EGFR* has long been known as a driver oncogene in non-small-cell lung cancer (NSCLC). NSCLC tumors with *EGFR* mutations generally have low PD-L1 expression levels [71], but *EGFR* can also be an intrinsic mechanism, causing the tumor to express high levels of PD-L1 [72]. This suggests that immune checkpoint inhibitors may be less effective in *EGFR* mutation-positive NSCLC tumors, even if PD-L1 is highly expressed. In the CheckMate 057 trial comparing nivolumab with docetaxel as a second-line therapy for non-squamous NSCLC, docetaxel tended to have better outcomes in *EGFR* mutation-positive patients [73]. Similarly, in the OAK trial comparing atezolizumab and docetaxel in previously treated NSCLC patients, docetaxel tended to be more effective in patients with *EGFR* mutations [7]. Myeloid cells are abundant in *EGFR*-mutated lung adenocarcinomas [74]. Lung adenocarcinoma cell lines with *EGFR* mutations are also known to recruit regulatory T cells (Tregs) as a result of high C-C motif chemokine 22 (CCL22) expression, creating a suppressive tumor-immune microenvironment [75]. The balance of PD-1 expression between effector and Tregs can reportedly affect the therapeutic efficacy of an anti-PD-1 antibody [76]. For this reason, anti-PD-1 antibodies may cause hyperprogressive disease in some cases [77]. The TATTON trial combining osimertinib and durvalumab was conducted, but the increased incidence of interstitial lung disease meant that the safety profile was not met [78].

In *KRAS*-mutated lung adenocarcinomas, multiple immune-cell types are expanded, including CD8+ T cells, Tregs, and myeloid cells [74]. KRAS also induces an inflammatory-tumor microenvironment [79]. Myc has been reported to cooperate with Ras to escape immunity and drive lung-cancer carcinogenesis by successfully exploiting inflammation [80]. Specifically, Myc drives the expression of CCL9 to recruit macrophages, and also suppresses T-cell and natura-killer (NK)-cell recruitment by interleukin (IL)-23 [80]. Myc can affect cancer-cell metabolism, by, for example, promoting glycolysis and augmenting PD-1^+^ Tregs with abundant lactic acid [81]. *STK11* mutations are frequently found as inactivators of tumor-suppressor genes in NSCLC, and encode liver kinase B1 (LKB1), a protein that suppresses cell growth and metabolism. Loss of *STK11/LKB1* resulted in an increase in C-X-C motif ligand 7 (CXCL7), granulocyte colony-stimulating factor (G-CSF), and IL-6, recruiting neutrophils with T-cell-suppressive effects in a mouse model of *KRAS*-driven NSCLC [83]. Furthermore, the authors reported that inactivating mutations in *STK11/LKB1* led to decreased PD-L1 expression in the mouse model and patient tumors [83]. Thus, genomic aberrations in STK11/LKB1 have been reported to mediate a less-immunogenic-tumor microenvironment and be a major cause of resistance to anti-PD-1 antibody treatment in NSCLC patients [84]. Numerous other oncogenes and tumor-suppressor genes can influence the immune landscape of tumors [21,85]. In this article, we are focusing on the genes in the Wnt/β-catenin pathway which are among them.

### 3.2. Wnt/β-Catenin Induces T-Cell Exclusion

Tumor-intrinsic Wnt/β-catenin signaling was identified as a significant mechanism for establishing a T-cell-excluded tumor microenvironment. Wnt/β-catenin can induce the expression of activating transcription factor 3 (ATF3), resulting in the reduction of CCL4 in a mouse model of melanoma [17]. CCL4 is essential for the recruitment of dendritic cells. Specifically, Basic Leucine Zipper ATF-like Transcription Factor 3 (Batf3)-driven CD103^+^ dendritic cells are required for effector T-cell trafficking via the production of C-X-C motif chemokine ligand 9/10 (CXCL9/10) [86].

β-catenin-mediated immune evasion associated with T-cell-excluded phenotypes has been reported in various cancer types, suggesting a link between de novo resistance mechanisms and immune checkpoint inhibitors. In colorectal cancer, genomic and immunohistochemical results using The Cancer Genome Atlas (TCGA) data have been reported [87]. In other cancers, an association between β-catenin and immune exclusion in the tumor microenvironment has been reported in head and neck cancer [88], bladder cancer [89], adenoid cystic carcinoma [90], ovarian cancer [91], hepatocellular carcinoma [82], and NSCLC [92].

### 3.3. The Wnt/β-Catenin Pathway and Immunoediting in Cancer

In addition to the decreased production of CCL4 via ATF3, Wnt/β-catenin signaling in cancer cells affects immunoediting in a variety of other ways [19]. In NSCLC, activation of the Wnt/β-catenin pathway is associated with a higher tumor-mutation burden [92,93]. This indicates that cancer cells with Wnt/β-catenin activation have high immunogenicity. Direct associations of the mutated β-catenin gene with specific cancer antigens recognized by T cells have also been reported in melanoma [94]. Hence, cancer cells with tumor-intrinsic β-catenin-pathway activation may require immunoediting in order to survive.

First, the β-catenin pathway in cancer cells can affect tumor-associated macrophages (TAMs). The activation state of macrophages changes continuously, and TAMs are classically classified into M1 and M2 subtypes [95]. M1-like TAMs have antitumor effects, and M2-like TAMs can directly or indirectly suppress antitumor immunity. TAM-derived IL-1β can induce the phosphorylation of GSK-3β, resulting in stabilization of β-catenin in colon cancer [96]. Moreover, β-catenin activated by TAM-derived IL-1β can stabilize Snail, thereby further activating macrophages to produce IL-1β [97]. These findings suggest that β-catenin can mediate interactions between cancer cells and TAMs.

β-catenin activation in cancer cells was also associated with Treg infiltration [98]. Conversely, one report stated that β-catenin suppresses Tregs [99], causing there to still be controversy over whether β-catenin can stimulate Tregs or not [18]. In melanoma, IL-10 expression was associated with β-catenin activation [100]. IL-10 production is induced by β-catenin/TCF binding to the IL-10 gene promoter, and inhibits dendritic-cell maturation [100]. β-catenin activation in cancer cells results in dendritic-cell impairment.

In addition, β-catenin signaling in cancer cells produces an immunosuppressive tumor-microenvironment through metabolism. β-catenin signaling can also upregulate monocarboxylate-transporter (MCT1) and pyruvate-dehydrogenase-kinase-1 (PDK1) expression levels [31]. This causes glucose metabolism in cancer cells to shift from oxidative phosphorylation to aerobic glycolysis, resulting in lactate production and a more acidic tumor microenvironment. Lactate can suppress activation of T cells and dendritic cells, as well as induce M2-like macrophages, vascular endothelial growth factor (VEGF) and hypoxia inducible factor-1α (HIF-1α) [101]. Aerobic glycolysis and tumor growth can also induce a hypoxic tumor-microenvironment, which itself inhibits cytotoxic T-cell functions and leads to immune-checkpoint-inhibitor resistance [102]. Proteomics analysis in melanoma revealed that higher oxidative phosphorylation and lipid metabolism could elevate antigen presentation, and was associated with responses to anti-PD-1 immunotherapy [103].

## 4. Overcoming Immune-Checkpoint-Inhibitor Resistance with Wnt/β-Catenin Signaling

### 4.1. Combination Therapy with Chemotherapy and Immune Checkpoint Inhibitors

The combination of immune checkpoint inhibitors and chemotherapy can reportedly enhance therapeutic efficacy, especially in NSCLC [9,10,11,12,13]. Cytotoxic chemotherapy can result in immunogenic cell death, promote antigen presentation by dendritic cells [104], and eliminate myeloid-derived suppressor cells (MDSCs) [105] and Tregs [106]. Radiotherapy similarly induces immunogenic cell death, and is expected to enhance the efficacy of immune checkpoint inhibitors, either alone or in combination with chemotherapy [107,108,109]. Chemotherapy and radiotherapy are expected to promote antigen presentation and T-cell recruitment in tumors with active Wnt/β-catenin signaling. There have been no reports examining the effect of chemotherapy combined with immune checkpoint inhibitors in β-catenin-activated cancers. However, the results of chemotherapy combined with immune checkpoint inhibitors in NSCLC suggest that there may be additive but not synergistic effects. This means that although they may inhibit cancer progression or control tumor burden in the short term, they do not seem to increase the proportion of patients who achieve a long-term response to immune checkpoint inhibitors. Comparing the results of clinical trials of pembrolizumab alone with those of pembrolizumab combined with chemotherapy in non-squamous NSCLC patients, the data suggest that the combination with chemotherapy was able to reduce disease progression in the early treatment period, but has made little difference to long-term survival [110,111,112]. Cancers with activated β-catenin signaling are considered “immunologically cold” tumors, and often have low or negative PD-L1 expression [92]. Therefore, the additive effect of chemotherapy is likely to be temporary. Lung cancers with β-catenin-pathway activation have been shown to be resistant to chemotherapy, and patients have a poor prognosis with or without adjuvant chemotherapy [47,51]. Similarly to *STK11* genomic aberrations, activation of the β-catenin pathway may be a prognostic factor rather than a predictor of response to combination therapy of immune checkpoint inhibitors and chemotherapy [113]. In a mouse model of melanoma, using an anti-PD-1 antibody plus an anti-CTLA-4 antibody had no effect on tumors with β-catenin activation [17]. The combination of other immune checkpoint inhibitors and molecular targeting-agents would likely be similarly ineffective.

Activation of the β-catenin pathway is frequently observed in hepatocellular carcinoma [114], and is linked to resistance to immunotherapies [82]. However, another report states that the combination of an anti-PD-L1 antibody with an anti-VEGF antibody showed efficacy, regardless of β-catenin-pathway activation [115]. In liver tumors, the tumor microenvironment is highly glycolytic, and Tregs express PD-1 more frequently than effector T cells [81]. Additionally, anti-PD-1 antibodies are ineffective when PD-1 is more highly expressed in Tregs than in effector T cells [76]. These data suggest that the therapeutic strategy of suppressing Tregs with an anti-VEGF antibody in combination with an anti-PD-L1 antibody may be effective in the treatment of hepatocellular carcinoma.

### 4.2. Neoadjuvant Treatment Strategies

If it is difficult to treat cancers with activated β-catenin signaling long-term, by combining chemotherapy and immune checkpoint inhibitors, a possible strategy may be performing surgical resection while the lesion is shrinking to some extent. There have been numerous clinical trials of neoadjuvant treatment methods for NSCLC, albeit in the resectable stage rather than for advanced cases. In the CheckMate 816 trial, surgical resection after four courses of nivolumab and chemotherapy resulted in pathological complete response in 24% of stage IB to IIIA resectable-NSCLC patients [116]. Results from the phase 2 NADIM trial have also been published, but the relationship between activation of the β-catenin pathway and survival or pathological response-rates has not been published [117]. For reference, of the 26 patients who achieved a complete pathological response, one patient had disease progression, and this patient had an *EGFR* mutation. Of the seven patients with major pathological response (≤10% viable tumor cells), one had mutations in *STK11* and had disease progression [117]. These findings suggest that even in a resectable neoadjuvant setting, it may be difficult to expect long-term efficacy using a combination-therapy approach with chemotherapy and an immune checkpoint inhibitor, as oncogenic signals can lead to tumor-intrinsic immune evasion. In addition, surgical resection was canceled in five of the forty-six patients who received neoadjuvant therapy in the phase 2 trial, and in 15.6% patients in the nivolumab-plus-chemotherapy group in the phase 3 trial [116,117]. Reasons for cancellation varied, but included those who could not undergo surgery because of disease progression. When neoadjuvant treatment is administered on tumors with active β-catenin signaling, the pathological response rate and percentage of patients who could undergo surgery should be confirmed in the future.

### 4.3. Combination Therapy with Inhibitors of Wnt/β-Catenin Signaling and Immune Checkpoint Inhibitors

The simplest way to suppress Wnt/β-catenin signaling is to use specific inhibitors. Developing drugs that modulate Wnt/β-catenin signaling has been a focus for decades, but to date has not been clinically successful [118]. In syngeneic mouse-tumor-models, β-catenin inhibition with CTNNB1 Dicer siRNA (DCR-BCAT) significantly increased T-cell infiltration, and potentiated the sensitivity to immune checkpoint inhibitors [119]. DCR-BCAT is an intravenously delivered lipid-nanoparticle containing oligonucleotide, which selectively silences *CTNNB1* [120]. A number of non-coding RNAs (ncRNAs) can reportedly affect β-catenin levels in hepatocellular carcinoma, but have not reached clinical application [121]. In ongoing clinical trials, several Wnt/β-catenin inhibitors are also being investigated, in combination with immune checkpoint inhibitors (Table 2). One of the most promising Wnt/β-catenin signaling inhibitors is the porcupine (PORCN) inhibitor [122]. PORCN is indispensable for Wnt binding to its receptor Frizzled, which triggers Wnt/β-catenin signaling. In a phase-1 study in solid tumors combining WNT974, a PORCN inhibitor, and spartalizumab, an anti-PD-1 antibody, stable disease was reported in 53% of patients who were primary refractory to prior anti-PD-1 antibody treatment [123]. Another candidate drug is DKN-01, which is a humanized IgG4 monoclonal antibody that binds and neutralizes circulating Dickkopf-1 (DKK1). Secreted DKK1 is characterized as an inhibitor of the Wnt/β-catenin-dependent (canonical) pathway, but an activator of Wnt/β-catenin-independent (non-canonical) pathway signaling [124]. Because DKN-01 does not directly inhibit the Wnt/β-catenin pathway, it can reportedly enhance innate immunity rather than alter the T-cell-excluded tumor microenvironment to become an inflamed state [125]. The efficacy of DKN-01 was enhanced in combination with an anti-PD-1 antibody in a murine model of melanoma and metastatic breast cancer [125]. A phase-1b study of DKN-01 in combination with pembrolizumab in advanced esophagogastric cancer showed objective response rates of 50% in DKK1-high and 0% in DKK1-low patients [126]. These trials are being conducted on a variety of cancers.

## 5. Conclusions

Identifying and overcoming immune-checkpoint-inhibitor resistance mechanisms is a central concern in cancer therapy. Depending on the specific biology of the tumor, sensitivity to immune checkpoint inhibitors can vary. Tumor-intrinsic resistance mechanisms can affect host immunity in various ways. Wnt/β-catenin signaling is one of these tumor-intrinsic resistance mechanisms, and is responsible for the excluded phenotype. Activation of β-catenin induces ATF3 expression, which decreases CCL4 and results in decreased recruitment of DCs. Without DCs, lymphocytes do not infiltrate into the tumor. β-catenin in tumors can also create an immunosuppressive environment by interacting with TAMs, affecting Tregs and altering metabolism in the tumor microenvironment. To overcome the immune evasion caused by β-catenin, the current combination-therapy approaches of anti-PD-1/PD-L1 antibodies with chemotherapy or anti-CTLA-4 antibodies may not be sufficient. The long-term outcomes of using a neoadjuvant anti-PD-1/PD-L1 antibody with chemotherapy are also questionable in NSCLC patients with β-catenin-induced immune evasion. Preclinical studies have shown that inhibiting Wnt/β-catenin signaling can enhance the efficacy of immune checkpoint inhibitors. Clinical development of agents that directly inhibit the Wnt/β-catenin pathway has historically been difficult, but their use in combination with immune checkpoint inhibitors is currently being examined, with the results eagerly awaited.

## Figures and Tables

**Table 1:** Genetic aberrations promoting immunoediting in non-small-cell lung cancer (NSCLC).

Gene	Genetic Aberration	Resulting Immune Editing	Reference
EGFR	Mutation	PD-L1↑, Tregs	[70,73]
		Myeloid cells↑	[72]
KRAS	Mutation	Myeloid cells↑, CD8 T cells↑, Tregs↑	[72]
MYC	Amplification	Macrophages↑, NK cells↓, T cells↓	[78]
		Tregs↑, Lactic acid↑	[79]
STK11/LKB1	Loss	Neutrophils↑, T cells↓, PD-L1↓	[80,81]
β-catenin target genes	Amplification	DC↓	[82]

PD-L1, programmed death-ligand 1; Tregs, regulatory T cells; NK, natural killer; DC, dendritic cells.

**Table 2:** Current clinical trials combining a Wnt/β-catenin signaling inhibitor with an immune checkpoint inhibitor.

Drug	Target	ICI Agent	Cancer Type	Trial Phase	Clinical Trial
LGK974	PORCN	PDR001	Solid tumors	I	NCT01351103
ETC-1922159	PORCN	Pembrolizumab	Solid tumors	I	NCT02521844
CGX1321	PORCN	Pembrolizumab	Solid/GI tumors	I	NCT02675946
RXC004	PORCN	Nivolumab	Solid tumors	I	NCT03447470
RXC004	PORCN	Nivolumab	Colorectal cancer	II	NCT04907539
DKN-01	DKK1	Pembrolizumab	Esophagogastric cancer	I	NCT02013154
DKN-01	DKK1	Nivolumab	Biliary tract cancer	II	NCT04057365
DKN-01	DKK1	Atezolizumab	Esophagogastric cancer	I/II	NCT04166721
DKN-01	DKK1	Tiselizumab	Esophagogastric cancer	II	NCT04363801

PORCN, Porcupine; DKK1, Dickkopf-1; GI, gastrointestinal.

## Data Availability

No new data were created or analyzed in this manuscript. Data sharing is not applicable to this article.
